# A Metapopulation Model for Preventing the Reintroduction of Bovine Viral Diarrhea Virus to Naïve Herds: Scotland Case Study

**DOI:** 10.3389/fvets.2022.846156

**Published:** 2022-07-18

**Authors:** Gavrila A. Puspitarani, Rowland R. Kao, Ewan Colman

**Affiliations:** ^1^Roslin Institute, Royal (Dick) School of Veterinary Studies and the Roslin Institute, University of Edinburgh, Midlothian, United Kingdom; ^2^Unit Veterinary Public Health and Epidemiology, University of Veterinary Medicine, Vienna, Austria

**Keywords:** metapopulation model, bovine viral diarrhea, prevention strategies, animal movements, Scotland, endemic livestock disease

## Abstract

**Background:**

Bovine viral diarrhea (BVD) virus is one of the most problematic infectious pathogens for cattle. Since 2013, a mandatory BVD eradication program has successfully reduced the number of infected cattle living on Scottish farms; however, England remains at high prevalence and presents a risk to Scotland through animal movement.

**Methods:**

We analyze cattle movements in the UK from 2008 to 2017 and recorded incidence of BVD in Scotland from 2017 to 2020. To simulate BVD reintroduction into Scotland, we developed an epidemiological model that combines transmission between cattle and animal movements between farms. A total of four control strategies were implemented in the model: no intervention, import restriction, targeted vaccination, and combined strategy.

**Results:**

During the course of the eradication scheme, movements into Scotland became increasingly distributed in regions close to the England–Scotland border. The prevalence of BVD in this region decreased at a slower rate than the rest of Scotland during the eradication scheme. Our model showed that the change in the prevalence is expected, given that the change in the patterns of movement and if vaccination is targeted to the border areas that decrease in the prevalence will be seen throughout the whole of Scotland.

**Conclusion:**

Scottish farms are susceptible to BVD virus reintroduction through animal imports from non-BVD-free nations with farms in border areas being the most vulnerable. Protecting the border regions provides direct and indirect protection to the rest of Scottish farms by interrupting chains of transmission.

## Introduction

Bovine viral diarrhea is known to cause severe economic loss in cattle farming. Its economic impact is characterized by poor milk quality and quantity, development of mucosal disease, reduced pregnancy rate, congenital defects, and fetal death (1). Because the disease is not zoonotic, the concern around BVD is solely in the economic losses incurred and the reduction in animal welfare.

Bovine viral diarrhea virus (BVDV) infections are either transient or acute, and very few persist within the host (2). BVDV transmission occurs through close contact with infected animals and typically leads a transient infection (TI) lasting around 3 days (3). If a cow is infected during gestation period, they may produce persistently infected (PI) offspring. Though TIs are believed to play a role in transmission, PI animals are immunotolerant and act as the main reservoir of infection responsible for maintaining the virus within the herd (4, 5). In a country with no BVD control program in place, it has been shown that around 1%−2% of calves born will be PIs (6). Therefore, the presence of one or more PI animals within a herd is the major concern for cattle keepers to control BVD.

Various countries have shown that it is economically profitable for the cattle industry to control and eliminate BVD. Financial losses due to BVD have been reported for a number of reasons including reduced milk production, high feed conversion rate, animals culled, additional veterinary visits, treatment, vaccination, and laboratory testing (7, 8). For instance, after the BVD eradication program in Norway from 1992 to 2002, it was estimated that dairy and beef farms could have gained up to 29 million NOK (7, 9). In the United Kingdom (UK), annual losses from BVD were estimated to range from 5 to 31 million pounds, whereas the loss for Scottish cow-calf herds cost £37 per cow per year (10, 11).

The Scottish government, in collaboration with the private sector, developed a BVD eradication scheme which started in 2010 then become mandatory in 2013 (12). The Scottish scheme adapts the combined approach of removing PI animals followed by herd vaccination after clearing to avoid reinfection (13). By 2019, 90% of herds were BVD-free (14). Meanwhile, England and Wales have remained with only a voluntary program (15, 16), with an estimated up to 30% of farms in Wales remaining infected (6, 17) and 0.2%−3.1% PI prevalence within the infected herds (18).

Scotland may soon find itself in a situation where BVDV is largely eliminated from the country, with only sporadic outbreaks. However, it would remain susceptible to the reintroduction of the disease through importation of infected animals from non BVD-free regions. Infected animals known as Trojan cows, dams carrying an infected fetus, where the pregnancy status at the time of selling is often unidentified and the BVD status is negative, present a unique risk (19). Even at very low PI prevalence, due to the large volume of trade, there is still the possibility for the transfer of Trojan cows into farms with high standards of disease management (20). Therefore, studying animal movement from non-BVD free regions is critical to understanding the risks posed in introducing animals.

Here, we investigate the impact of animal importations and movements into Scotland on the potential for disease reintroduction. We first describe the effect of the eradication scheme on the pattern of animal movement and disease incidence in Scotland. We then develop a metapopulation model, accounting for both the spread of infection within-herds as well as the dynamics of cattle moving into and between regions of Scotland. We use this model to study the transmission of BVD in Scotland through cattle movement to show the level of exposure to the risk of BVD reintroduction. Finally, we consider several mitigation strategies to combat these risks.

## Methods

### Data

Livestock case data from 2008 to 2017 for BVD in Scotland were provided at request by the Scottish livestock traceability research team (ScotEID). Since 2001, the UK government has made it mandatory that all cattle births, deaths, arrivals from overseas, and exits, and movements between premises are recorded and held on a database known as the Cattle Tracing System (CTS) (21). CTS is a valuable source of recorded cattle movement data in the UK which is very useful for various epidemiological analysis including modeling disease spread (22). Location coordinates of all farms in the UK are provided using the British National Grid reference system. The data provided for this study were anonymized to prevent personal information from being disclosed. Specifically, farm locations have been changed by a distance of up to 10 km, and movement events have been aggregated to a monthly scale.

A movement is defined as a change in farm location over a 1-month time period; thus, short stays in intermediary locations such as markets, or movements that end in slaughterhouses are removed from the data. Short visit is thought to give less opportunity for infectious contact and the spread of slowly transmitting diseases (23).

BVD prevalence data was obtained from Scottish Government situation reports (not publicly available). From a sample of farms across Scotland, the number of PI cattle alive, the number of farms that were given not-negative status, and the number of breeding herds were provided at county level. We obtained 44 of these reports from August 2017 to April 2021.

**Figure 1:**
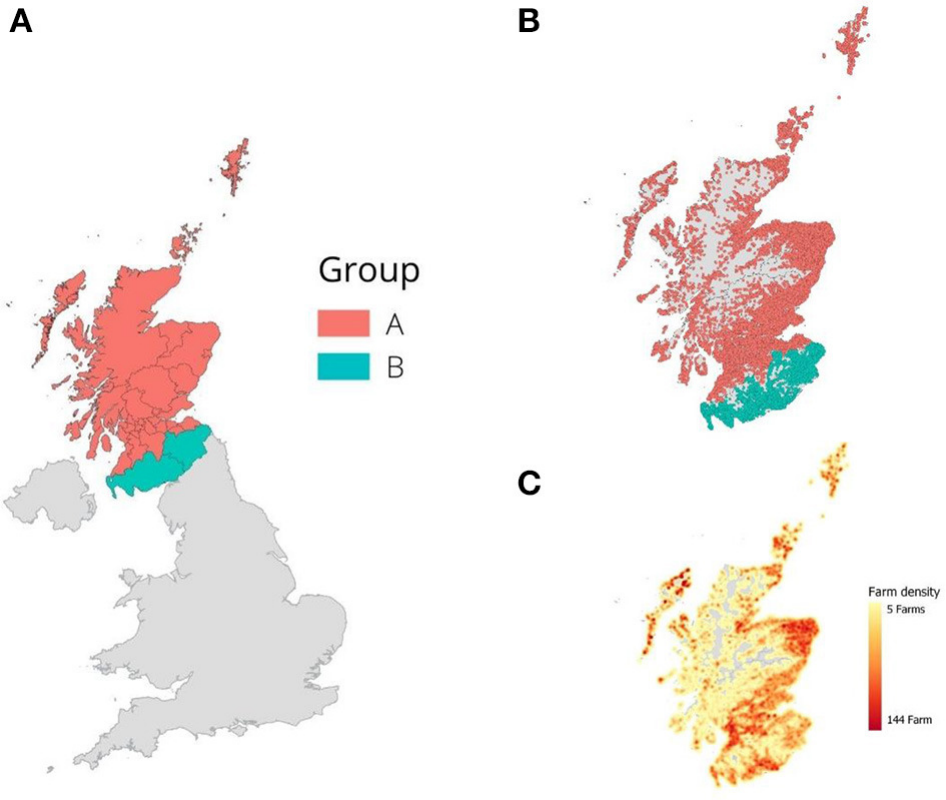
Group division of local authorities in Scotland. **(A)** UK map and categorizing local authorities in Scotland into two, Group A (*G*_*A*_) and B (*G*_*B*_). **(B)** Distribution of farms. **(C)** Heat map of farm density pointing that animal holdings are heavily distributed in the south and east of Scotland.

We classified farms into three groups, denoted by *G*, based on their geographical location (Figure 1). Group A, *G*_*A*_, is the set of farms that are in Scotland, excluding the two local authority regions that border with England. Group B, *G*_*B*_, is the set of farms that are situated in local authority regions that are bordering with England: Dumfries and Galloway, and Scottish Borders. All premises that are not located in Scotland are considered as an import Group, denoted as *G*_*I*_. We select only farms that are located in each of these regions by mapping its coordinates in the Great Britain shapefile, a digital vector storage for storing geometric location (24), with QGIS application.

### Models

Our model is a stochastic compartmental model, which accounts for two levels of mixing: first, within each farm where the dynamics of transmission are described by disease compartments, and second, the disease can be carried from one farm to another when an infected cow changes its location.

#### Animal Transition Between Disease Compartments

**Figure 2:**
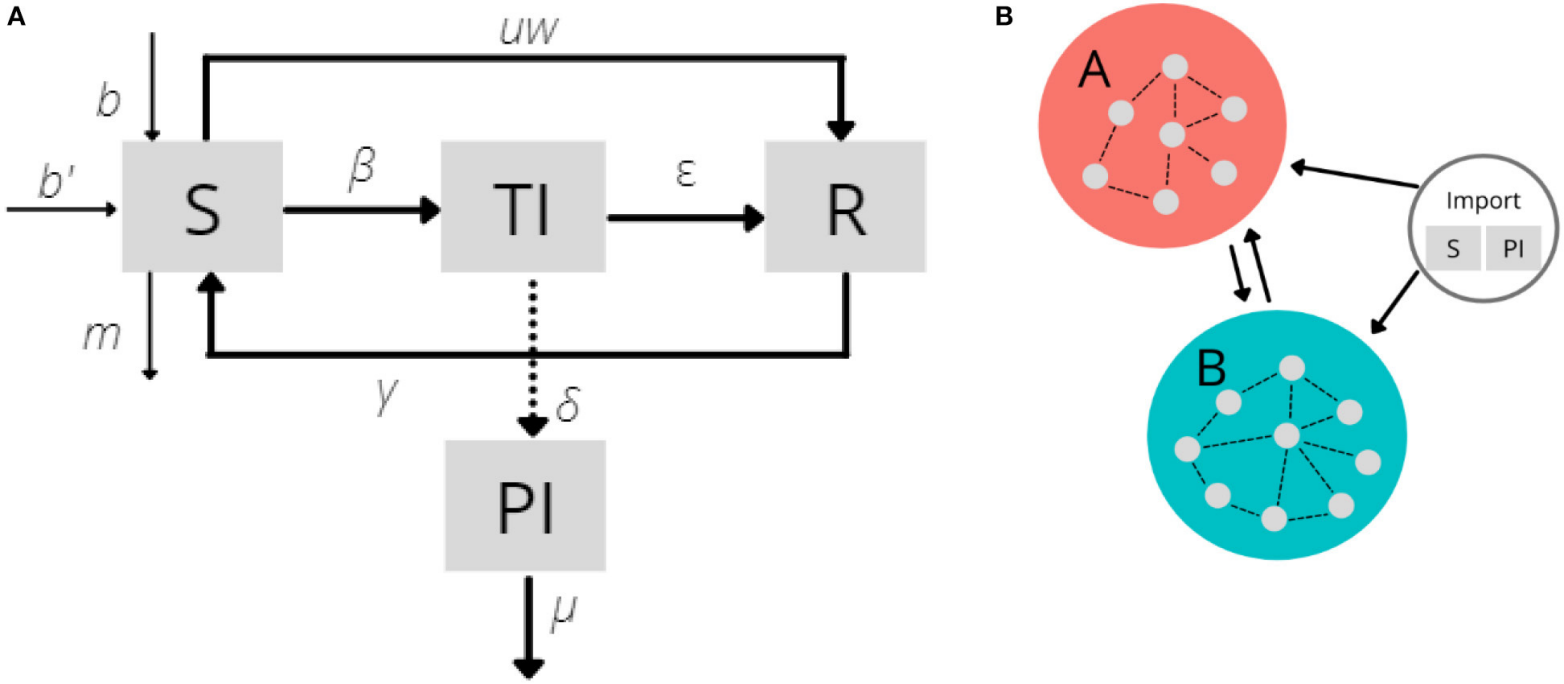
Flow diagram of animal's transition between compartments and group. **(A)** Compartment model of animal transition. Consist of susceptible (S), transiently infected (TI), persistently infected (PI), and recovered (R). **(B)** Metapopulation dynamics between groups. Dots inside the circle represent farms and its connections. New animals introduced from imports are susceptible and persistently infected.

The infection dynamics in a farm are based on a compartmental model in which the infection status of each animal with respect to BVDV is represented in a number of compartments, as illustrated in Figure 2A:

- Susceptible (S). The susceptible compartment (S) represents cattle who have not been exposed or are serological negative. At the initial stage of the model, all of animals in Scotland are susceptible.
- Transiently infected (TI). Once a susceptible animal is infected with BVDV, the animal becomes transiently infected and has the possibility of giving birth to a newly persistently infected calf. In our model, we considered TI animals as entirely uninfectious.
- Persistently infected (PI). The spread of BVD will occur if there is at least one persistently infected animal present within the herd. The PI animal is a lifelong carrier and shedder of the virus and will be noticeably smaller than other calves of the same age. If any PI is detected, they should be culled immediately. They are also unlikely to contribute to the birth of PI calves.
- Recovered (R). Animals may gain immunity and enter the recovered compartment. This can happen by natural infection or though vaccination. In our model, the animals may lose their immunity with time and re-enter the susceptible compartment.

### State Transitions

The model consists of nine state transitions listed in Table 1. Susceptible animals may give birth to susceptible calves at rate of *b*. We set a removal rate, *m*, from susceptible animals to represent animals going to slaughter, exported out of the region, and natural death. To have a stable susceptible population, we propose to have *b* = *m*. Susceptible animals become TI at rate of β, the transmission rate. The rate of TI animals giving birth to PI animals is δ, and thus, δ × *TI* is the number of newly born PI calves which are then introduced to the PI compartment per unit time. In addition to giving birth to PI calves, TI animals give birth to non-PI calves at rate *b*′ which will directly enter the S compartment (20).

**Table 1:** Transitions in the within-herd model.

**Event**	**State transition**	**Rate**
Birth to susceptible	∅ → **S**	**b**.**S**
Death of susceptible not related to BVDV infection	**S** → ∅	**m**.**S**
Infected susceptible become transiently infected	**S**→**TI**	$\frac{\beta .S.PI}{S+TI+PI+R}$
Birth of persistently infected calves	∅ → **PI**	***δ***.**TI**
Removal of persistent infected animals	**PI** → ∅	***μ***.**PI**
Transiently infected animals recover	**TI**→**R**	***ε***.**TI**
Recovered animals lose immunity	**R**→**S**	***γ***.**R**
Birth of non-persistent infected calves from transiently infected cows	∅ → **S**	**b^′^.TI**
Susceptible become immune due to vaccination	**S**→**R**	**u**.**w**.**S**

*The rate shown is the number of animals that experience the transition in each time step*.

The presence of PI animals within the herd is the main driver of BVDV spread to the susceptible animals. Both newly born and newly imported PI animals will remain within the PI compartment and will be removed at rate of μ per unit time. A fraction of ε animals from the TI compartment will gain natural immunity per unit time. In practice, we set value of ε = β to ensure that the duration of transient infection is neither too short to eliminate the possibility of further transmission, yet short enough that natural recovery does happen within a realistic time frame.

Animals who gained immunity either through vaccination or natural infection will eventually revert to being susceptible. The available BVD vaccine generally prevent infection, especially for transplacental infections of the fetus, although the protection seems to be low (25). A fraction γ × *R* of recovered animals reenters the susceptible compartment per unit time, where γ is the recovery rate. When the vaccination strategy is in place, the model will transfer *u* × *w* × *S* animals from S compartment directly to R compartment. We define *u* as the vaccination coverage (the probability of receiving the vaccine) and *w* as vaccine efficacy (the probability that it will provide protection).

Since we would like to study the disease spread after no BVDV present in Scotland by having imported animals as the main risk of reintroduction, we will assume all Scottish farms are initially BVDV-free. Thus, all cattle in Scotland are initially in S compartment and TI animals do not die from BVDV. This is consistent with observations that both virus biotypes lead to the development of specific antibodies but not death (26).

#### Movement Between Farms

Movements between farms are represented as a network: each farm is a node and a fixed probability is associated with every pair of nodes defines the edges. The parameters of movements between groups are based on the historical cattle movement data obtained from CTS. As the model moves forward in every time step *t* of 1 month, cattle move from their current farm to a destination farm with the probability associated with the edge between them. Nodes are organized into two groups, *G*_*A*_ and *G*_*B*_, assigned by their location (Figure 2B). The third group or imported group, *G*_*I*_, represents animals living outside Scotland. Import movement is defined as the arrival of cattle from non-Scottish farms to farms in Scotland that both buy and sell (meaning we excluded farms that took animals from outside of Scotland and have zero outdegree). There are 8 types of movement: those from: (i) *G*_*A*_ → *G*_*A*_; (ii) *G*_*A*_ → *G*_*B*_; (iii) *G*_*B*_ → *G*_*A*_; (iv) *G*_*B*_ → *G*_*B*_; (v) *G*_*I*_ → *G*_*A*_; (vi) *G*_*I*_ → *G*_*B*_; (vii) *G*_*A*_ → *G*_*I*_; (viii) *G*_*B*_ → *G*_*I*_, movements between farms in *G*_*I*_ are not considered in the model.

In the intergroup movements, we allowed movement of any animal between *G*_*A*_ and *G*_*B*_, however, from *G*_*I*_ → *G*_*A*_ and *G*_*I*_ → *G*_*B*_, we introduced only susceptible and PI animals. We controlled the number of PIs through setting a prevalence of imported animals (27). Each movement moves only one animal at time *t*.

#### Group Network Flow

The CTS data provide the number of movements each month between every pair of farms in the UK. For each pair of movements, *G* → *G*′, we calculate the movement probability as $P\left(G\to G^{\prime }\right)=\frac{s}{n}$, where *s* is the number of movements *G* → *G*′ from 2008 to 2017, and *n* is the total number of all movements from all group pairs during the same period. A fixed number of movements occur throughout the time period of the model. The time of each movement is selected uniformly at random from within the chosen period. We generate movements by randomly selecting an origin group and a destination group according to these probabilities, and then randomly sampling both the origin and destination nodes within the selected origin and destination groups. This process is repeated for every scheduled movement event at time *t*. The value of movement probabilities can be seen in Table 2.

**Table 2:** Movement probabilities between groups calculated from observed cattle movement data between 2008 and 2017.

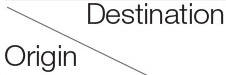	**G_A_**	**G_B_**	**G_I_**
*G*_*A*_	0.66	0.03	0.03
*G*_*B*_	0.11	0.08	0.04
*G*_*I*_	0.04	0.01	N/A

#### Vaccination

It has been suggested that to prevent the emergence of new PI animals, the population immunity should be 100% (13); however, in reality, vaccination does not always provide total protection and it is unlikely that all susceptible animals will be vaccinated due to practical and financial limitations. We propose strategies of vaccination coverage, denoted as *u*(*t*), that represent the proportion of susceptible individuals being vaccinated at time *t* (28) where *u* = 0 means there is no vaccination in place and *u* = 1 suggest total coverage of vaccination. The proportion of cattle successfully immunized at time *t* is *w* × *u*(*t*).

#### Scenarios

There were three proposed scenarios to address the research questions. The baseline scenario is the situation when there is no further control or restriction of cattle movements imposed in the country. The import restriction scenario (sc1) is where we simulate a reduction of 50% in the proportion of imports that go to *G*_*A*_. Specifically, we define the import restriction as allowing only a fraction of movements from *G*_*I*_, denoted as *I*, to *G*_*A*_. This case generates a new probability of importation: $P^{\prime }\left(G_{I}\to G_{A}\right)=P\left(G_{I}\to G_{A}\right)\times I$, where *P* and *P*′ denote the probability that a movement will be of the type selected and the modified probability, respectively. Since reducing imports to one specific group is assumed to create a reduction of the animal supply in the other groups, we compensate the reduction from *G*_*I*_ to *G*_*A*_ by adjusting the probability of trade between the other groups.

First, we define the new probably, *P*′, of movement from $G_{I}\;to\;G_{B}\;as:\;P^{\prime }\left(G_{I}\to G_{B}\right)=P\left(G_{I}\to G_{B}\right)+\left(1-I\right)P\left(G_{I}\to G_{A}\right)$, as the consequence of import restriction to *G*_*A*_. We also compensate the movement probability of domestic trade by setting $P^{\prime }\left(G_{B}\to G_{A}\right)=P\left(G_{B}\to G_{A}\right)+\left(1-I\right)P\left(G_{I}\to G_{A}\right)$ and $P^{\prime }\left(G_{A}\to G_{B}\right)=P\left(G_{A}\to G_{B}\right)-\left(1-I\right)P\left(G_{I}\to G_{A}\right)$. Note that this leaves the total number of cattle imported to the country, the total number moving into *G*_*A*_, and the total number moving into *G*_*B*_ unchanged.

Next, the rate of export from *G*_*A*_ and *G*_*B*_ is also modified to keep the total number of exports out of the country unchanged by: $P^{\prime }\left(G_{A}\to G_{I}\right)=P\left(G_{A}\to G_{I}\right)+\left(1-I\right)P\left(G_{I}\to G_{A}\right)$ and $P^{\prime }\left(G_{B}\to G_{I}\right)=P\left(G_{B}\to G_{I}\right)-\left(1-I\right)P\left(G_{I}\to G_{A}\right)$. Finally, movements within each group will not experience any adjustment, meaning $P^{\prime }\left(G_{A}\to G_{A}\right)=P\left(G_{A}\to G_{A}\right)$ and $P^{\prime }\left(G_{B}\to G_{B}\right)=P\left(G_{B}\to G_{B}\right)$, so the probabilities will remain the same. In brief, import restrictions are put in place to reduce the proportion of imported animals to *G*_*A*_ to 50% of the initial amount and then imports and domestic movements between the other groups are adjusted to compensate for the reduction in trade. By having this approach, the total import value to Scotland is unchanged but where the import arrives (*G*_*A*_ or *G*_*B*_) is changed.

Scenario sc2 considers the impact of targeted vaccination. We target 80% of total animals in *G*_*B*_ to observe the direct and indirect protection to animals in *G*_*A*_. In every 12th time step, representing annual vaccination rollout, we schedule the vaccination of *u* animals in the S compartment in *G*_*B*_ and allow the movement of vaccinated animals from *G*_*B*_ to any other groups. The duration of vaccination is happened only one time (1 month) for every year (i.e., 12 time steps). The final scenario, sc3, combines the movement restriction, sc1, and a targeted vaccination strategy, sc2. We summarized all of our scenarios in Table 3.

**Table 3:** Scenarios description.

**Scenario**	**Description**
Baseline	No control or restriction
1	Import restriction: reduce 50% of import proportion to *G*_*A*_ and allocate the proportion to the other group
2	Targeted vaccination: vaccinate 80% of animals in *G*_*B*_ only and occur one time in every 12 time steps
3	Combined strategy: combining import restriction and targeted vaccination

### Model Implementation

#### Parameters

All parameters' values can be seen in Table 4.

**Table 4:** Summary of all parameter values.

**Parameter**	**Definition**	**Values**	**Units**	**References**
β	Transmission rate	0.5	Monthly	(29)
ε	Recovery rate	0.189	Monthly	
δ	Rate of TI giving birth to PI animal	0.0094	Monthly	
μ	Removal rate of PI animal	0.083	Monthly	
γ	Rate of losing immunity	0.0833	Monthly	
*w*	Vaccine efficacy	80	%	(30)
*b*	Birth rate	0.0246	Monthly	Equation: (31)
*b'*	Rate of TI giving birth to normal calf	0.0098	Monthly	Equation: (31)
*m*	Removal rate	0.0246	Monthly	Equation: (31)
*P*_*I*_	PI prevalence	2%		(18, 32)
*I*	Import restriction	50%		
*u*	Vaccination coverage	80%	Year	
	Years of simulation	120	Month	
	Repetition	100		

We adapt transmission rate (β) from the study conducted by Han et al. (29) for beef herds, which also represent the most common herd type in Scotland (33). We choose to have a slower transmission rate due to the farming common practice to not directly introduce new animal with the existing herds. Note that this represents transmission from PI cattle; transmission from TI cattle is not included in the model. For the birth (*b*) and removal (*m*) rate value, we took the recorded birth and removal data from 2008 to 2017 which accounted for 25.6% on average from the total moves. We took this average number as removal rate and convert the rate using the standard method described by (31). The rate is calculated by *b*/*m* = –ln(1 – 0.256)/12 = 0.0246 per animal per month. The rate is applied for both the birth and removal rate.

To calculate the rate of new-born PI (δ), we referred to (31) by first calculating fertility reduction $\left(a\right)=\ln\left(\frac{X}{\theta X}\right)/t$. In this equation, *X* is the proportion of new calves born each year and θ is proportion of reduction, which is 0.2 (31). The birth rate for normal calves (calf that is healthy and not infectious) from TI animals is *b*−*a*, denoted as *b*′. Variable *X* followed the Scottish birth rate mentioned above, so *a* = ln(1.256/1.0512)/12 = 0.0148 per animal per month. Therefore, *b*′ = 0.0246−0.0148 = 0.0098 per animal per month. Fertility reduction (*a*) includes embryonic death, abortus, still birth, and PI animals as forms of losing productive offspring. Therefore, δ = *a* − *abortion*, where proportion of abortion is 0.12–0.14 from total pregnancy (34, 35). We then obtained δ = 0.0094 per animal per month.

Animals in the TI compartment will remain infected for certain period and moved to R compartment at rate ε as they developed immunity against the disease. TI animals are responsible for the production of new PIs, depending on the stage of gestation when infected, so if a dam gets infected at early stage of pregnancy, it may produce a PI calf (36, 37). The period that animals stay in the TI compartment represents the period for which an infected dam can produce a PI calf, not the duration for which TI can shed virus and infect other susceptible animals. Although TI also excrete virus, but very low rate (29, 38). While this model choice might miss the opportunity for TI to communicate the disease, we expect the effect to be small since outbreak tend to reach susceptible animals very quick due to PI high transmission rate. When the mandatory BVD eradication program reached its end, we assumed that the farmers are more relaxed to remove PI animals (μ), but not very long to retain the animals as they normally will die due to the absence of immune system (36, 38).

While we have used the best available literature to inform our choices for each parameter, but are aware of the uncertainty around these choices. We therefore undertake a sensitivity analysis, selecting a number of alternatives on the basis of their likely impact on our outcomes. Specifically for μ, when farmers are very active to detect and remove PI animals, it will lead to a higher removal rate (0.5), while if opted to keep infected animals for a very long time gives lower removal rate (0.0132). Similarly, we also tested whether TI animals take a shorter time to gain immunity (0.5) or longer time to enter recovered compartment (0.083). This additional analysis is provided in Supplementary Material.

We assume that the immunity will last for an average of 12 months, which was chosen based on the BVD vaccine recommendations (39, 40). So, choosing γ = 1/12 to ensure immune cattle will lose their immunity after an average time period of 12 months and return to the S compartment. The prevalence of imported PI animals (*P*_*I*_) is 2%, based on studied field PI and prevalence in Wales (18, 27). Several studies reported that the BVD vaccine efficacy gives around 80% protection (30, 41). The number of effectively vaccinated cattle, animals who successfully obtained protection against BVD, will then enter the recovered compartment after scheduled vaccination.

#### Initial Conditions

At the start of the simulation, all farms have 100 susceptible animals and no PI animals present. The model is run for 10 years over 100 iterations. We used the actual recorded total movements of 1,458,258 of events occurred throughout the time period of the model. There are 32,129 and 5,918 farms in *G*_*A*_ and *G*_*B*_, respectively. The model simulates the spread of BVD within the groups A and B, which are initially BVDV-free, which occurs after the introduction of at least one imported PI animal.

### Software

The statistical analysis, modeling, and plots were done using RStudio version 1.4.1103. The SimInf library was used to model the disease introduction (42).

## Results

### Cattle Movements and BVD Prevalence

**Figure 3:**
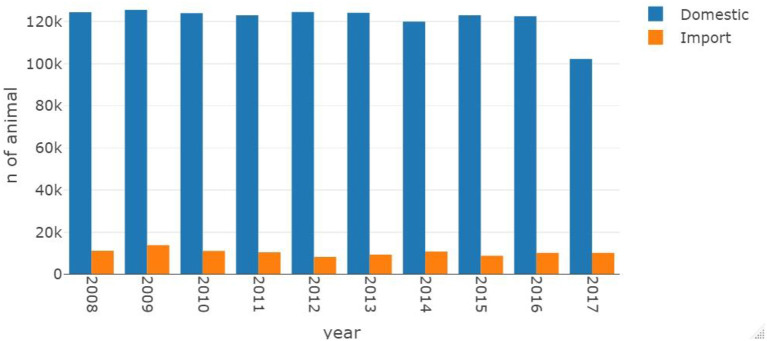
Movement frequency from 2008 to 2017 in Scotland. The color in blue showed domestic movement frequency, animals depart and arrive from farm in Scotland. While the orange showed import movement frequency, animals originated not from Scottish farms.

Cattle movement data from 2008 to 2017 (2017 data were incomplete) were processed to obtain animal movements from farm to farm and imports arriving at farms in Scotland. The frequency of movements within Scotland did not increase or decrease significantly during the course of the mandatory BVD eradication program (Figure 3). Similarly, the number of cattle being imported into Scotland did not change over this period.

**Figure 4:**
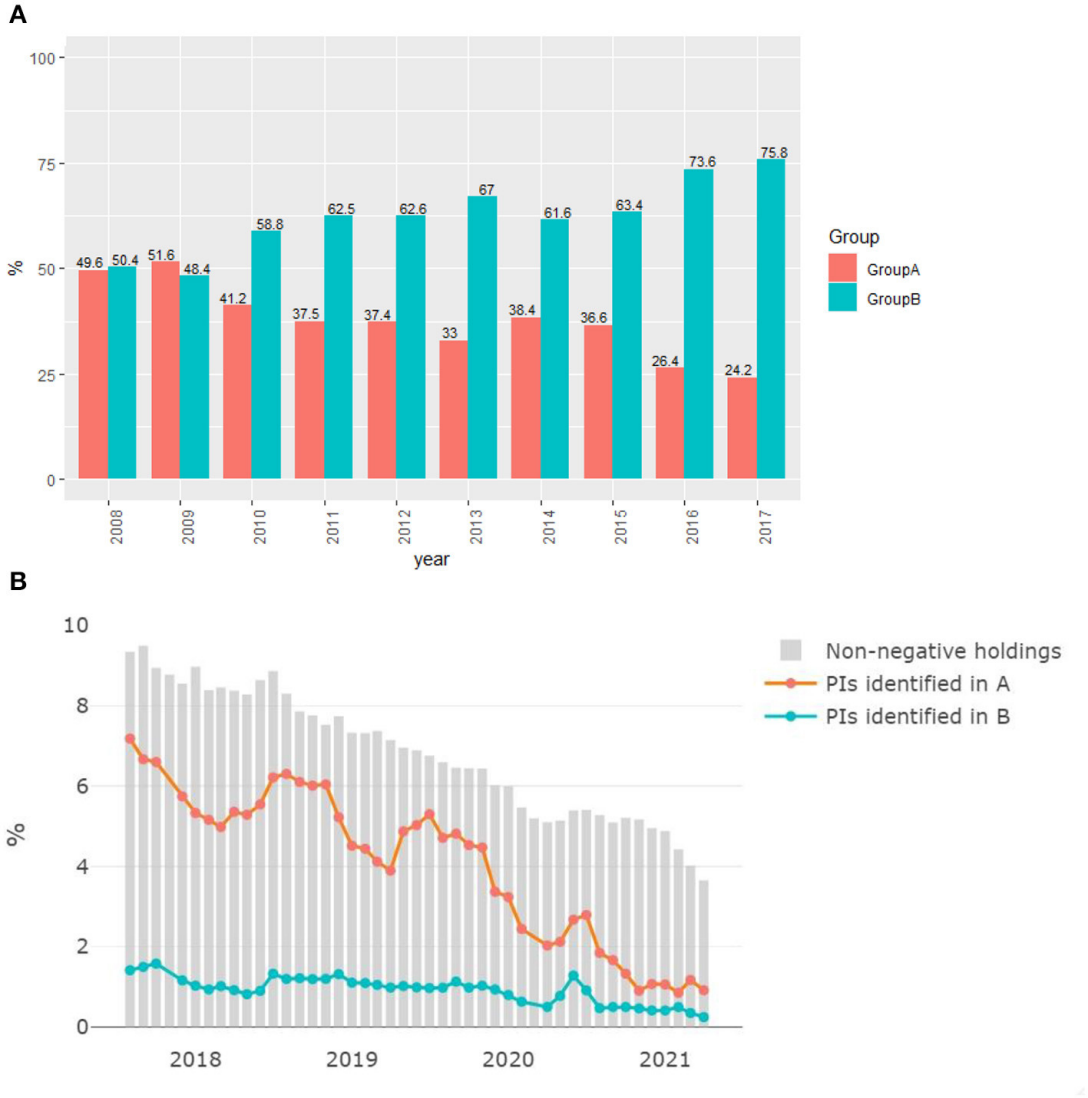
**(A)** Proportion of imports going to either *G*_*A*_ or *G*_*B*_. **(B)** Proportion of all non-negative holdings in Scotland is shown in bar graph. While the lines present the proportion of identified living PIs in both groups.

Farms in the region that shares a border with England, *G*_*B*_, received more imported animals than *G*_*A*_. Figure 4A shows the increase in import demand of *G*_*B*_ compared to *G*_*A*_ since 2009. By 2016, the total number of imported animals entering *G*_*B*_ is 150% higher compared to *G*_*A*_. Hence, throughout the duration of the eradication scheme, animals from outside of Scotland have become increasingly more likely to arrive in *G*_*B*_.

The number of identified PIs alive also depleted significantly from 2018 to 2021 in both *G*_*A*_ and *G*_*B*_ (Figure 4B). An outlier was removed from data (November 2017). As recently as 2021, the total number of PIs alive identified in Scotland ranged from 47 (April 2021) to 69 (May 2021), a reduction of 80% compared to 2017. The significant reduction is likely the result of successful enforcement by the Scottish government and compliance from Scottish farmers. By 2021, <5% of the total sample of farms in Scotland were stated as non-BVD-negative.

The number of PIs in *G*_*B*_ is higher than *G*_*A*_. This is due to the higher frequency of import animals coming to *G*_*B*_ than to *G*_*A*_. We also observe that the geographical distribution of BVD prevalence has changed over time, moving toward the English border; in 2017, 27% of the farms that were given non-negative status were in *G*_*B*_; by 2021, this number had risen to 35%. Given this trend, it is reasonable to suggest that the risk of BVD in Scotland comes almost entirely from imported animals.

### Modeled Control Strategies

**Figure 5:**
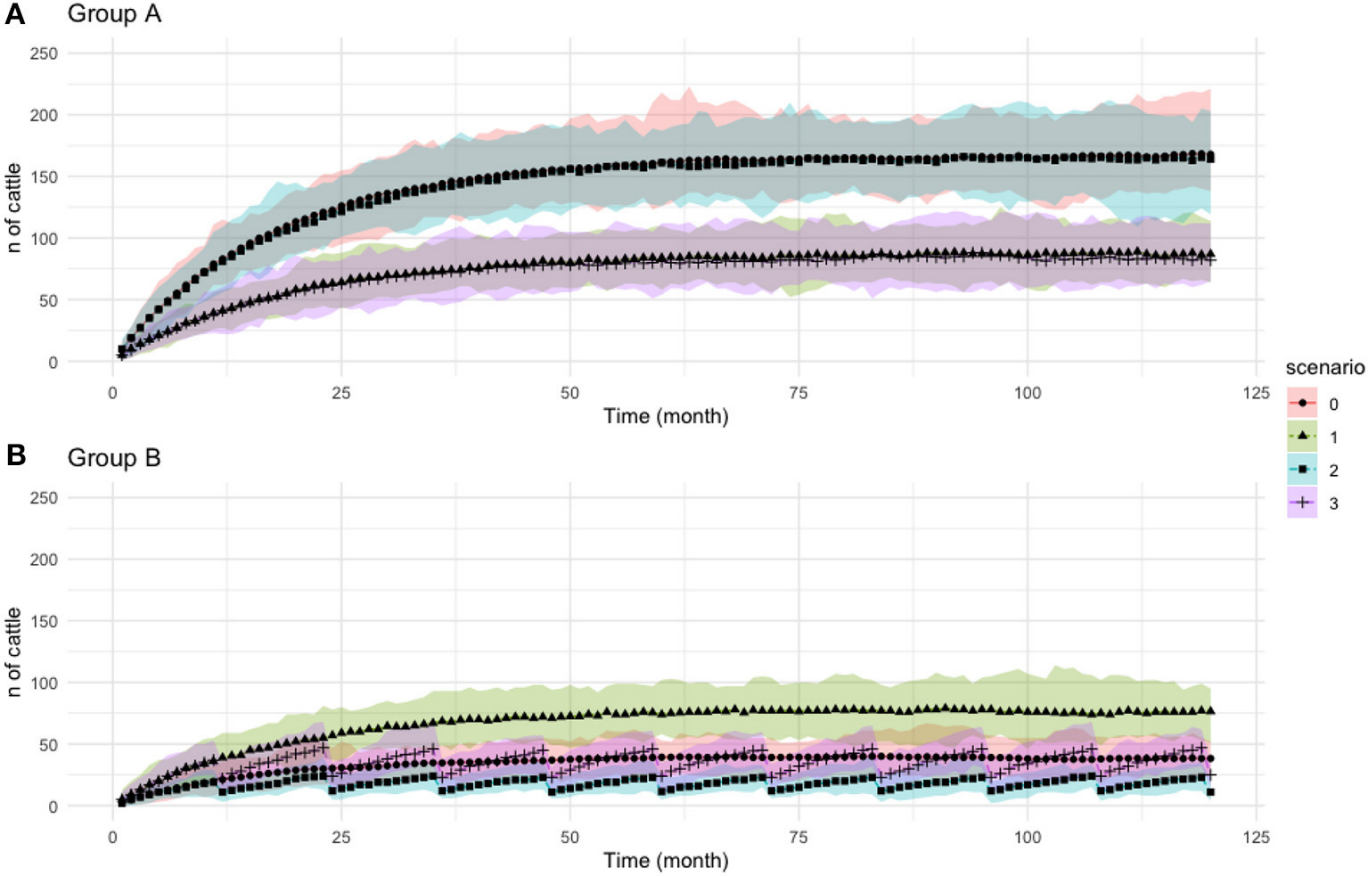
Population of persistently infected (PI) animals over time. **(A)** Population in Group A. **(B)** Population in Group B. Legend color and shape represent different scenarios for each group. For every colored shade presented the range of values obtained from 100 simulations, upper shade is the maximum value and lower shade is the minimum. Each shape in each scenario presents the mean value. Scenario 0 represent baseline scenario. The *y*-axis is the number of cattle and *x*-axis is monthly time step.

**Figure 6:**
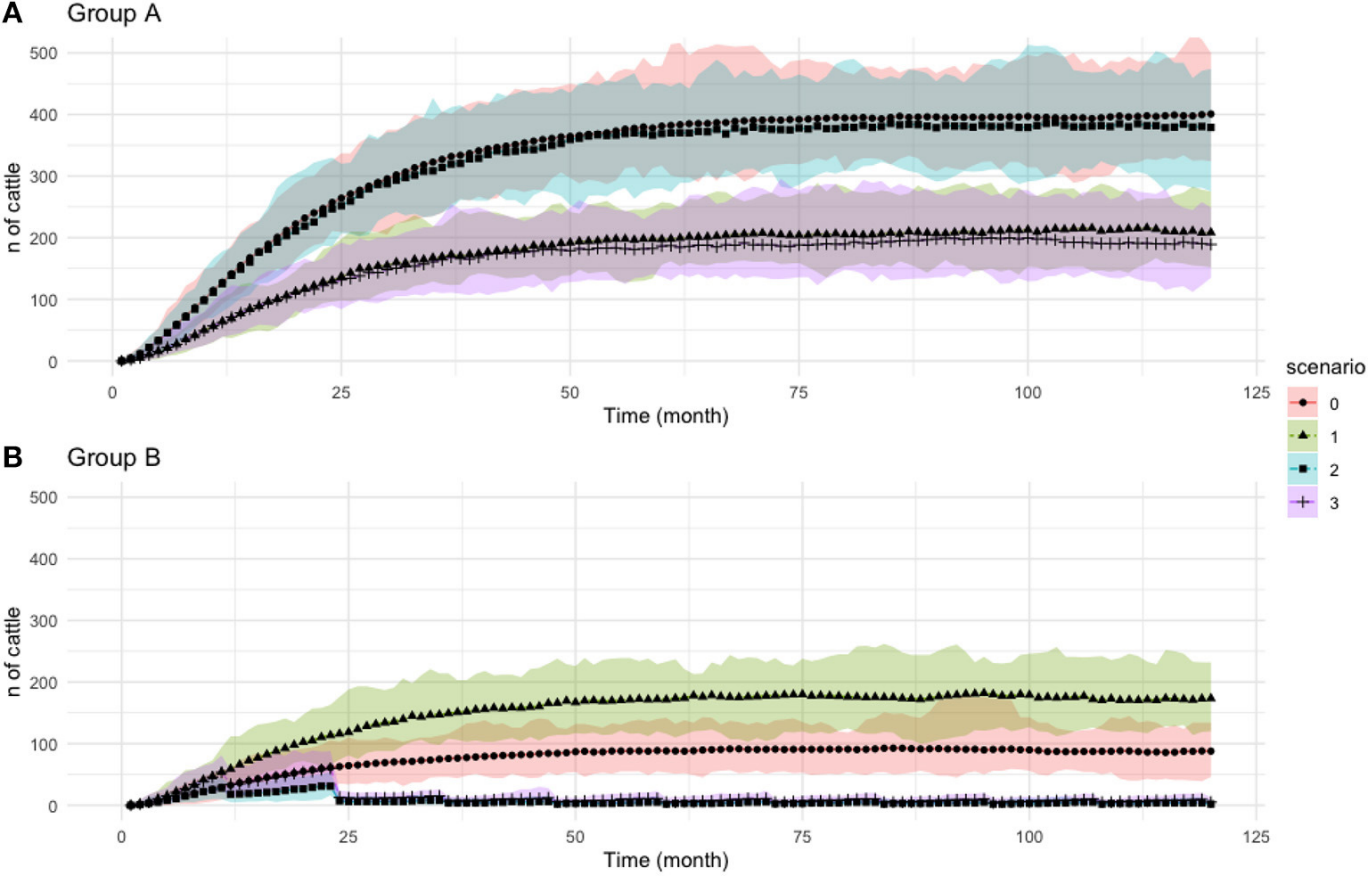
Population of transiently infected (TI) animals over time. **(A)** Population in Group A. **(B)** Population in Group B. Legend color and shape showed different scenarios for each group. Scenario 0 represent baseline scenario. The *y*-axis is the number of cattle and *x*-axis is monthly time step. Note that the axis scale in this figure is different from Figure 5.

**Figure 7:**
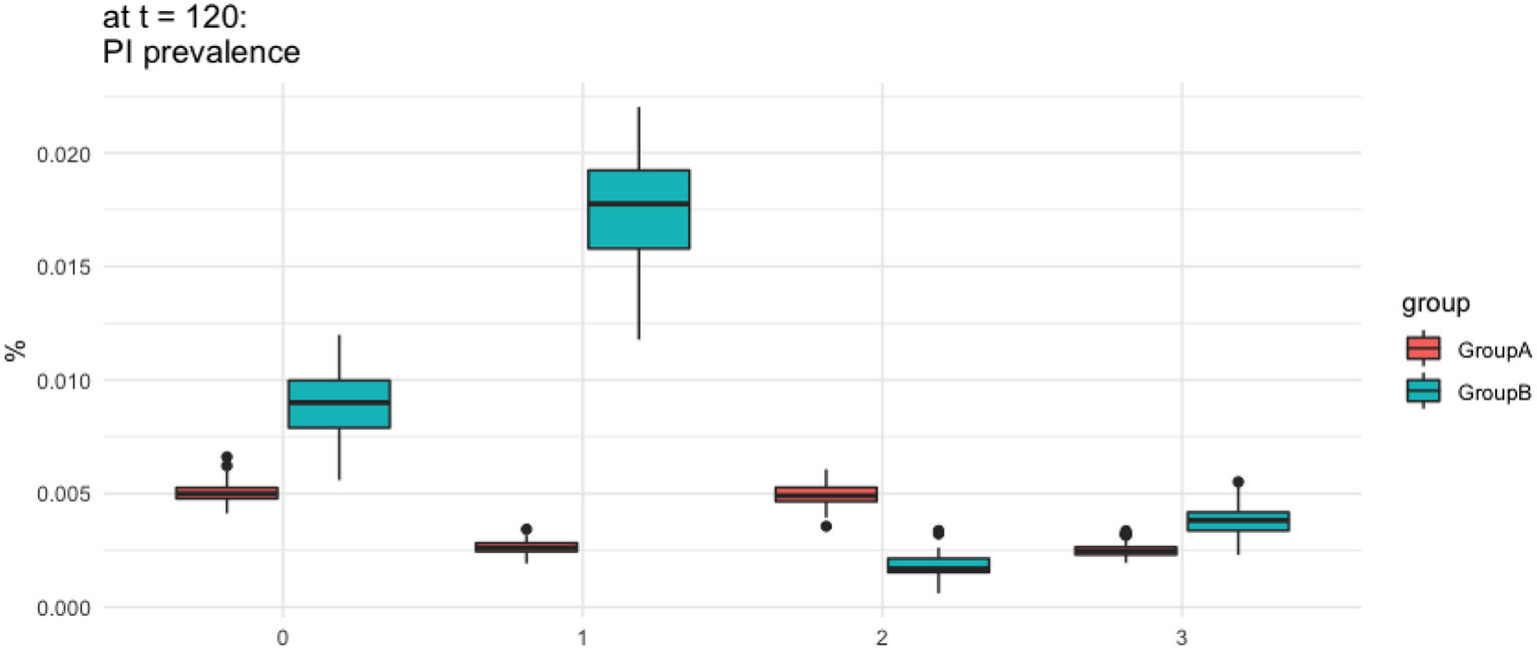
Prevalence of persistently infected animals. The *y*-axis presents the percentage of animals that are PI form the total cattle population in each scenario. Scenario 0 represent baseline scenario. This plot aims to compare the prevalence of PI in each type of scenario in each group at the end of simulation time (t = 120).

The result for PI and TI population over time is represented in Figures 5, 6, respectively, whereas PI prevalence at the end of simulation (*t* = 120) is illustrated in Figure 7.

#### Baseline Scenario (sc 0)

The model predicts that importing animals from a non BVD-free area, with the prevalence of 2%, leads to the growth of PI's population within all farms in Scotland (Figures 5A,B). The mean value of PI prevalence at the end of simulation time (*t* = 120) between groups *G*_*A*_ and *G*_*B*_ is significantly different (*p* < 0.05), with *G*_*B*_ being the highest (Figure 7).

#### Scenario 1 – Import Restriction (sc1)

Scenario 1 differs from the baseline by distributing a larger proportion of imports to *G*_*B*_ and a smaller proportion to *G*_*A*_, continuing the trend seen in Figure 4A. The population of PIs in *G*_*A*_ grows at a slower rate compared to *G*_*B*_ (Figure 5), which leads to a higher PI population in *G*_*B*_ compared to baseline. This situation in *G*_*B*_ occurs due to the increased number of imports to *G*_*B*_ that compensates for the reduced imports to *G*_*A*_.

At the end of the simulation (*t* = 120), the model shows more than 50% decrease in the prevalence of PI animals in *G*_*A*_ compared to the baseline. The prevalence of PI animals in *G*_*B*_ is 49% higher than in the baseline scenario. This suggests that the destination of imports into Scotland will ultimately change the direction of the situation significantly. This also illustrates how animal importations drive the PI prevalence.

#### Scenario 2 – Targeted Vaccination (sc2)

Scenario 2 differs from the baseline by providing vaccination to 80% of the animals on all farms in *G*_*B*_. As we would expect, the number of PI animals in the vaccinated region, *G*_*B*_, is now lower than the baseline scenario, as well as lower than the non-vaccinated *G*_*A*_. Unlike the baseline and sc1, PI population in *G*_*B*_ are stable, with the population maintained below 50 animals during the study periods. The wave-like pattern in Figure 5 results from the growth of PI from imports and birth followed by a sharp decline after vaccination (every 12 months) that effectively eliminates the TI population, seen in Figure 6, and therefore reduces new-born PIs.

Vaccination in *G*_*B*_ indirectly affects the risk to cattle in *G*_*A*_. The prevalence of PI animals in *G*_*A*_ at the end of the simulation (*t* = 120) was significantly lower in sc2 compared to sc0 (*p* < 0.05); however, it continued to grow throughout the modeled time period.

#### Scenario 3 – Combination (sc3)

The combination of movement changes from sc1 and vaccination from sc2 was shown to be overall the most effective at reducing the number of PIs. However, in *G*_*A*_, PI's population continued to increase albeit at a slower rate compared to all cases. While PI in *G*_*B*_ remained under control, the average population is higher than in sc2 (Figure 5). This is due to the increased number of imports to *G*_*B*_ that compensates for the reduced imports to *G*_*A*_.

The combination of import restriction and targeted vaccination showed to decrease PI's prevalence. The prevalence in *G*_*A*_ demonstrated a lower value compared to vaccination only (sc2; *p*-value < 0.05), which showed that adding targeted vaccination will ultimately increase the protection in *G*_*A*_ by having a lower PI population.

##### Sensitivity Analysis

We repeated the whole analysis under different combinations of parameter choice. These are included in the Supplementary Material S1. When ε is high (0.5), meaning TI animals quickly gained immunity, the overall PI prevalence is lower in this analysis compared to our main parameter due to the quick depletion of TI population. With lower ε (0.083), PI prevalence yield higher ε, especially for *G*_*B*_ in al scenarios. Similarly, with TI prevalence, lower and higher ε yield higher and lower prevalence, respectively, which reflect the percentage of animals that are PI.

Furthermore, when parameter μ is very high (0.5), meaning farmers can quickly detect and removed TI animals, the PI prevalence is lower compared to our initial parameter. However, when μ is very low (0.0132), where farmers choose to retain PIs at longer time, this leads to a very high percentage of PI in the total cattle population. This signifies that farmers decision to detect and remove infectious animals influences the disease dynamic. In general, the option in both parameters shows similar results in the pattern of PI prevalence which we conclude that the parameter in our analysis is sufficient to represent the disease dynamic.

## Discussion

We developed a metapopulation model to predict the situation of BVD in Scotland, a country in the final phase of its eradication program. The results presented here are strategic analysis of the possible prevention scenarios for BVDV reintroduction through importation, since Scotland's closest neighbors, England and Wales, have considerably higher prevalence and do not have a mandatory program. Our model is adapted to real movements and import conditions; thus, the result is to support decision-making related to control this disease. This model may also be incorporated to build further investigation of economic consequences for different scenarios and used to allow cost–benefit analysis of the most reasonable prevention measures.

Scotland has successfully eliminated PI animals by having the Scottish government involved in the eradication scheme. From the rapid decrease in the number PI cattle alive in Scotland, we believe that at the end of phase 5, Scottish herds will be approaching BVD freedom. The current PIs alive and their locations are publicly accessible in https://www.scoteid.com/scoteid/bvd_pi_locator provided by Scottish government as a form of transparency. However, the website only published list of PIs who are retained in a holding for more than 40 days. They do not disclose holdings who were quick to remove PI cattle from the herd. This form of publicity is thought to encourage farmers to quickly remove PI animals from their holding before suffering the social consequences of being disclosed.

Since the rapid depletion of PIs in Scotland, which acted as the main source of BVDV spread, importation will become the main threat of BVDV reintroduction to the country as has been suggested by Albrecht et al. (43). The newly purchased animals which have the potential to give birth to PI calves will pose a threat to susceptible animals, which then makes that the BVD-free herds are fully susceptible to BVDV reintroduction, especially where BVD vaccination is limited (13, 16, 44). However, animal trade across nations in the UK is inevitable, such as in the bordering areas where the border control between nations is absence. Similarly, trade within farms in Scotland is expected to remain the same regardless the movement controls implemented since phase 3, thus making Scottish farms vulnerable to disease reintroduction.

From our investigation of animal importation frequency to the nation, it shown that there was no difference of import frequency regardless of the movement restriction that has been implemented since 2013 (12). However, imported animals coming to Scotland become more likely to arrive in farms located in the regions that share a border with England, suggesting a changing trend of animal trade since 2008, and strongly suggesting that the BVD eradication scheme has driven an alternative behavior in animal trades. This is likely to be the reason why the geographical distribution of BVD cases has increasingly become concentrated in the border regions; a trend that suggests that elimination of the virus from this area may be more challenging than in the more northern regions.

Higher imports received by the border areas are due to the geographical location that is much closer to England, which makes the logistics for trading more convenient. Farmers also have the tendency to buy cattle based on their trust in the seller and reluctant to check BVD status for private trade (45). Another interesting reason is due to the political point of view, some farmers believe that it is unnecessary to have a different eradication scheme with the rest of the UK and view it as a separatist movement (15). It is well-known that Scottish farmers in the south of Scotland have closer relations with English farmers, both in commercial and in social aspects. It is very likely that farmers in the border areas are more relaxed to the regulation of the Scottish BVD eradication program.

In the disease spread model, we obtained an expected result when there is no control in place; the number of PI animals will be very likely to increase and threaten naïve herds. Although it might overestimate the prevalence of imported PIs to Scotland (since Scottish farmers are encouraged to buy from farms with high standards of biosecurity), this model illustrates the likelihood of new PI calves being born due to the presence of TI animals. At the end of study, the disease spread model without control yielded PI prevalence below 0.01%, which is lower than national PI prevalence in Germany, a country that is deemed to have a successful effort in controlling BVDV (46). However, our model do not account the factor of breeding seasonality where temperature and photoperiod during winter, for example, are shorter which decrease the fertility and calving rate of cows (47) that possibly implicate the PI prevalence. Prevalence among countries who are deemed to have successfully eliminated BVD is, in fact, considerably >0. For example, 0.9, 0.03, and 0.01% are the prevalence in Sweden, Ireland, and Germany, respectively (43, 48, 49). Thus, we consider the prevalence of 2% PIs randomly introduced to *G*_*A*_ and *G*_*B*_ to be adequate to initiate a BVD outbreak because it imitates the situation like the countries mentioned above.

As imported animals are the main source to trigger a BVD outbreak in Scotland, restricting the number of animals coming to the country will definitively decrease new-born PI calves. This is evidenced with the result from investigating the scenario where movement to *G*_*A*_was further restricted. Controlling imports will significantly decrease the PI prevalence and vice versa. Therefore, movement control of imported animals should be one of the approaches to control PI prevalence. Such control has been implemented since phase 4 (50), but only applies for trades to Scottish breeding herds. Trade to non-breeding herds is currently restricted, which could permit imported non-disclosed PIs to enter Scotland and cause local outbreaks, for instance, through over-the-fence contact. The non-mandatory BVD eradication program in England and Wales might bring disadvantages to Scottish farms, as farms are not compulsory to have a negative certificate to be able to trade.

In the most reasonable case, there is no deliberate trade of infected animals to BVD-free areas, but rather, the risk to introduce the so-called Trojan cows (19, 51, 52). The re-emergence of BVDV in a naïve population due to a Trojan dam had been reported by the German federal state of Saxon-Anhalt (43). A similar study conducted by Reardon et al. (52) stated that retainment of BVD-positive herds in Ireland was due to trade of Trojan dams between farms. The current available testing protocol is to test new-born calves or cattle by virus or viral antigen detection assay (RT-PCR, Ag-ELISA), however, infected calves *in utero* are unable to be detected with the current diagnostic tests (5). Failure to detect and control movement of Trojan cows will present an epidemiological risk.

Farms located in the border areas are found to have a higher risk of BVD virus introduction due to higher imports arrival, and, thus model's control strategy in targeted vaccination in high-risk area found to be beneficial to protect susceptible animals, by lower PI prevalence. The model exhibited not only direct protection to border areas but also provides indirect protection to the unvaccinated region by cutting off potential chains of transmission. The simulation result indicates that annual vaccination is not a tool to eradicate but rather to control disease (13, 50), where it has succeeded in reducing the number of PI animals but did not result in zero prevalence. A possible approach to annual booster vaccination roll out, after initial primary doses, can be administered the vaccine 7–28 days before the start of gestation period (39), around April to May, to protect transplacental infection and the birth of persistent calves.

Despite knowing that vaccination is able to protect naïve herds, some countries prohibit the use of vaccination and allowing only strict surveillance and biosecurity measures (16). In return, robust surveillance is needed to quickly detect and remove infected animals (2). The option for implementing vaccination or strong biosecurity measures in Scotland should be explored further in terms of efficiency, cost, and farmer's preference to give a better reasoning for the decision-maker. This study also showed that vaccination alone is not sufficient to control BVDV circulation in the herds, but it has to be followed by other control measure such as imports control and removal.

Commercially available BVD vaccines give a variety of protections, including fetal protection and cross-protection with different antigenic strains (41), suggesting that future modeling should take into account that vaccination might have different effectiveness. In the context of BVDV, the goal of vaccination is shifted from an eradication tool to be a concept of disease control and prevention. However, vaccination implies additional costs which should be evaluated against their expected benefits, including herd immunity and animal welfare (53, 54).

### Recommendation

As England and Wales are still in voluntary BVD eradication program, which considers as not an optimal effort to eliminate the disease (55) and PI prevalence remains relatively high, the study recommends that the existing BVD eradication program in Scotland be continued. Trade between countries, especially animals arriving in southern Scotland, should be given more attention due to the possibility of introducing animals with no status. Targeting vaccinations in southern Scotland would serve as protection for the rest of Scottish farms; however, those who will bear the costs should be considered.

The best recommendation is to eliminate BVDV from the country by accelerating the eradication program in England and Wales. Additionally, it is important to increase control measures for importing animals from high prevalence areas.

### Limitation

The study was assuming 0% prevalence of PI at the start of the model, which does not truly reflect the situation in each Scottish farm, so it is possible that the Scottish prevalence may have been underestimated. However, the true prevalence of infected animals imported to Scotland is unknown.

There were several simplifications within the model. We were using only a single vaccine with standard efficacy instead having different combinations of BVD vaccines. We also assume that all calves born are directly going into susceptible compartment, rather than having certain period for maternal antibodies.

The movement of cattle between farms was modeled using only the probabilities of movement between different regions. The simplicity of this framework provides a clear and straight-forward methodology to compare the effect of various movement restriction scenarios. The drawback of this approach is that it creates some unrealistic behaviors, most notably that animals move one at a time rather than in groups, and that repeated trade between the same buyer–seller combination occurs infrequently. However, these inaccuracies are likely to have only a small impact given the relatively low incidence of BVD (meaning that, even if moved in batches, only small numbers of infected cattle are likely to be moved at one time). While we are aware these network effects to spread of the disease between farms (56, 57), especially since repeated movements between farms likely mean that some farms are at inherently higher risk than others, the very coarse scale at which we make comparisons means that such differences are unlikely to make a qualitative difference to our observations.

Due to data limitations, we do not take into account type of farming practices, beef or dairy, which possibly have different variations. Other sources of variability such as biosecurity, testing, and social pressure were not taken into account in the model, which may act to increase or decrease the risk of reintroduction.

## Conclusion

In conclusion, the model was built to model the risk of reintroduction of BVDV due to importation and proposed several strategies to control the source of viral outbreaks. Scottish farms in the Anglo-Scottish bordering areas are more vulnerable to the disease reintroduction due to high number of import animals. The model showed import restriction followed by strong vaccination will be able to keep the prevalence of BVD very low or undetected. Interestingly, targeted vaccination for herds located in the border areas showed the capability to protect the remaining Scottish herds. This finding illustrates an approach to many other livestock infectious diseases which include animal movements for disease management. We suggest to take into account the social and economic perspectives for implementing prevention measures as form of collaborative approach.

This study present a modest idea of disease spread through animal movements, and thus, it is adjustable with data and variable for other countries and can be reused for similar purposes.

## Data Availability Statement

Cattle Tracing System data information to support this study are available from Animal Plant and Health Agency (APHA) which was obtained via a data sharing agreement. BVD incidence data is held by the Scottish Government and made available upon request. Disease spread simulation is available in the repository: https://github.com/EwanColman/BVD_sim_inf_model/blob/master/BVD_model.R.

## Author Contributions

GP conceived the main research idea, prepared the manuscript, designed the methodology with supervision and contribution from EC and RK. EC provided all the data and information related. RK and EC also provided critical revision of the manuscript. All authors contributed to the article and approved the submitted version.

## Conflict of Interest

The authors declare that the research was conducted in the absence of any commercial or financial relationships that could be construed as a potential conflict of interest.

## Publisher's Note

All claims expressed in this article are solely those of the authors and do not necessarily represent those of their affiliated organizations, or those of the publisher, the editors and the reviewers. Any product that may be evaluated in this article, or claim that may be made by its manufacturer, is not guaranteed or endorsed by the publisher.
