# Supplementary material for: A Metapopulation Model for Preventing the Reintroduction of Bovine Viral Diarrhea Virus to Naïve Herds: Scotland Case Study
Source: Front Vet Sci. 2022 Jul 18;9:846156. doi: 10.3389/fvets.2022.846156 (PMC9444324; doi:10.3389/fvets.2022.846156)
Supplement: Supplementary file 1 [file Data_Sheet_1.PDF]

Supplementary figures: prevalence of persistently and transiently infected animals.

Parameter ε: transiently Infected animals recovered.

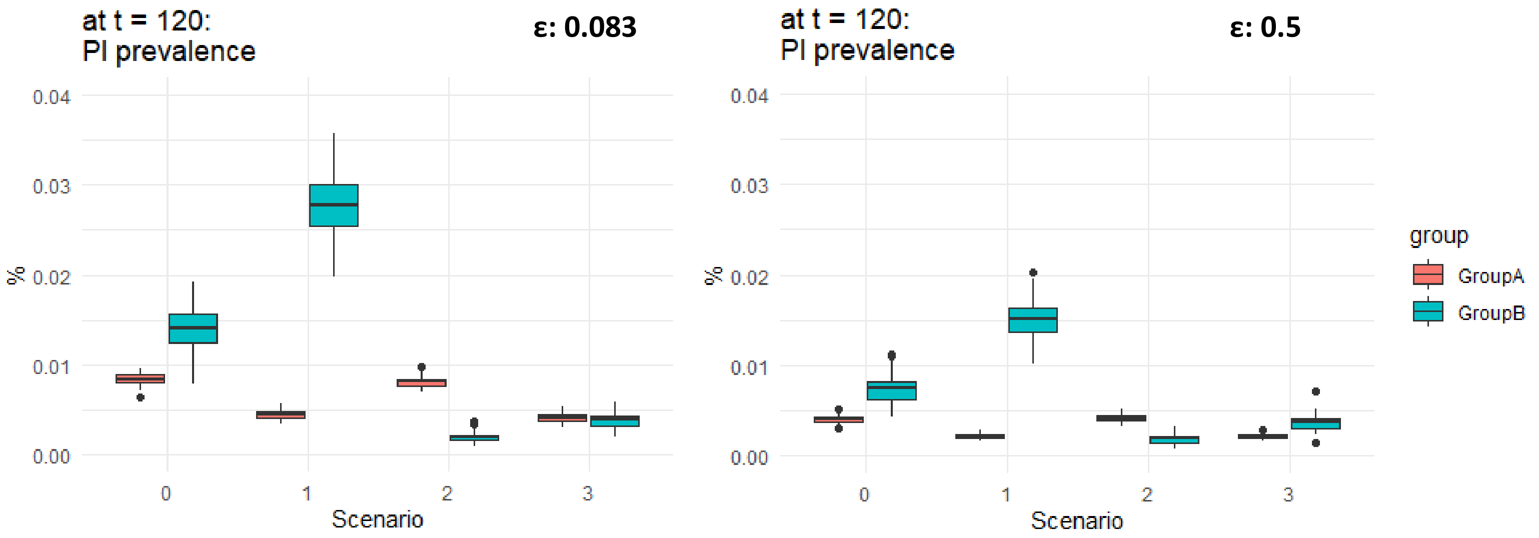

Supplementary 1. Prevalence of persistently infected animals with different ε value. The y axis presents the percentage of animals that are PI from the total cattle population in each scenario. Scenario 0 represent baseline scenario. This plot aims to compare the prevalence of PI in each type of scenario in each group at the end of simulation time ($t = 120$).

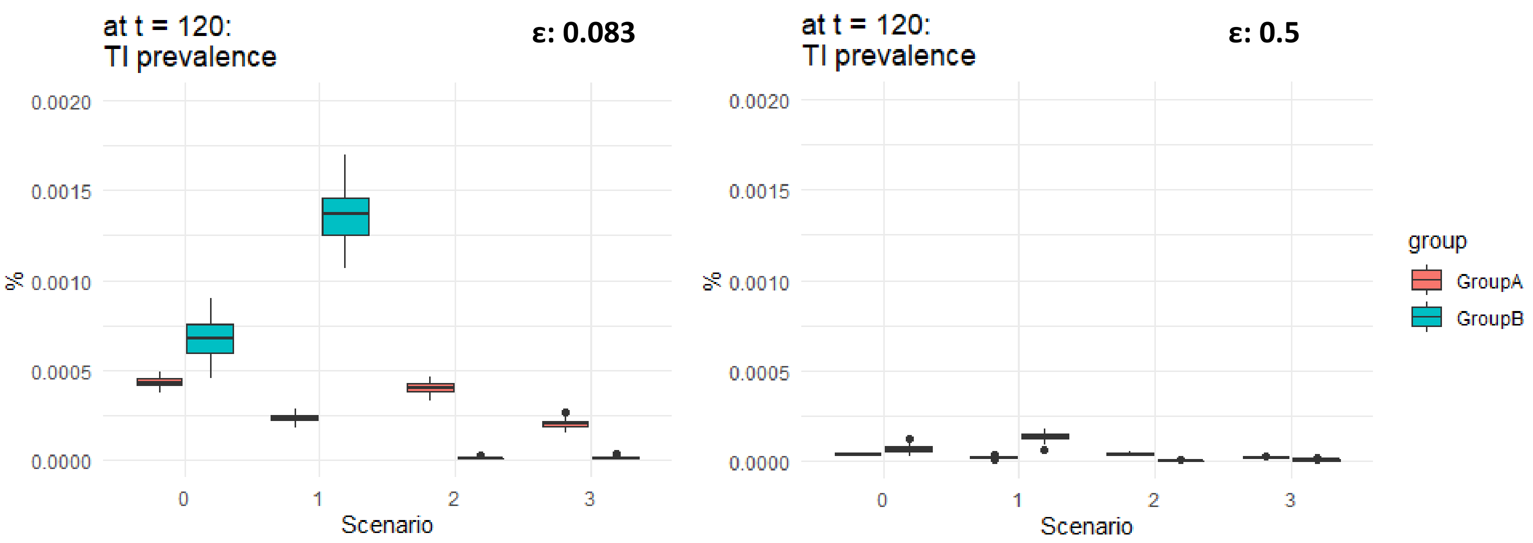

Supplementary 2. Prevalence of transiently infected animals with different ε value. The y axis presents the percentage of animals that are PI from the total cattle population in each scenario. Scenario 0 represent baseline scenario. This plot aims to compare the prevalence of PI in each type of scenario in each group at the end of simulation time ($t = 120$). Please note the y axis is slightly different from supplementary 1.

Parameter μ: removal rates of persistently infected animals.

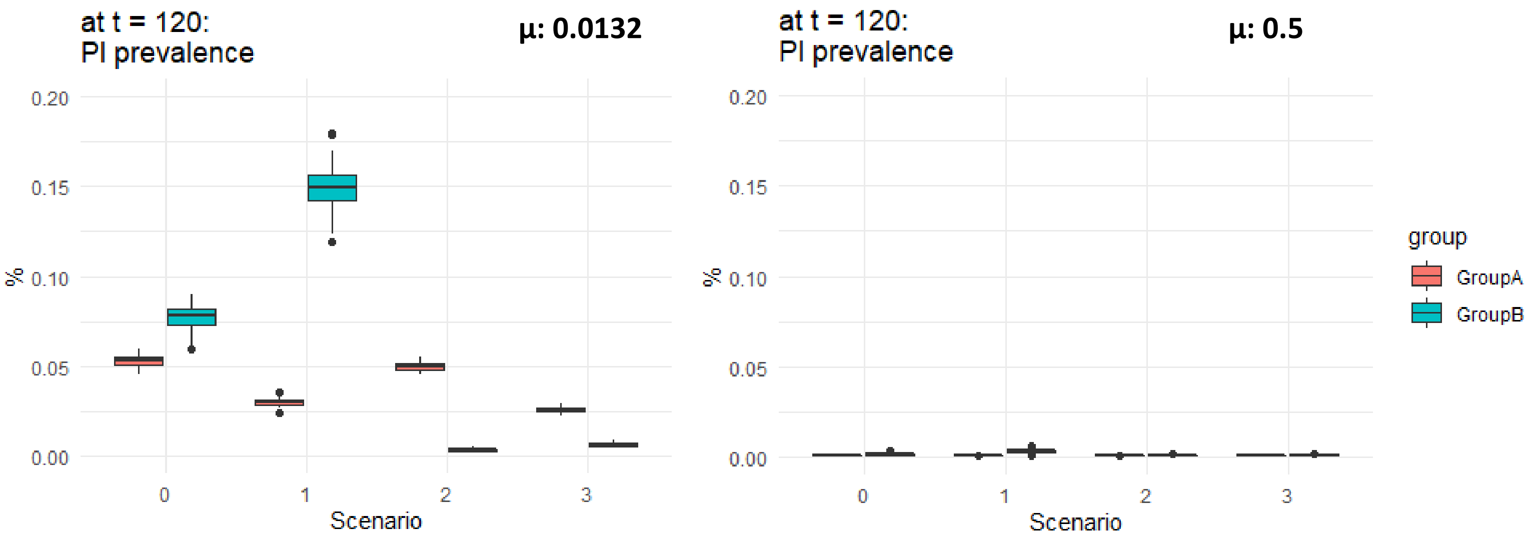

Supplementary 3. Prevalence of persistently infected animals with different μ value. The y axis presents the percentage of animals that are PI from the total cattle population in each scenario. Scenario 0 represent baseline scenario. This plot aims to compare the prevalence of PI in each type of scenario in each group at the end of simulation time ($t = 120$).

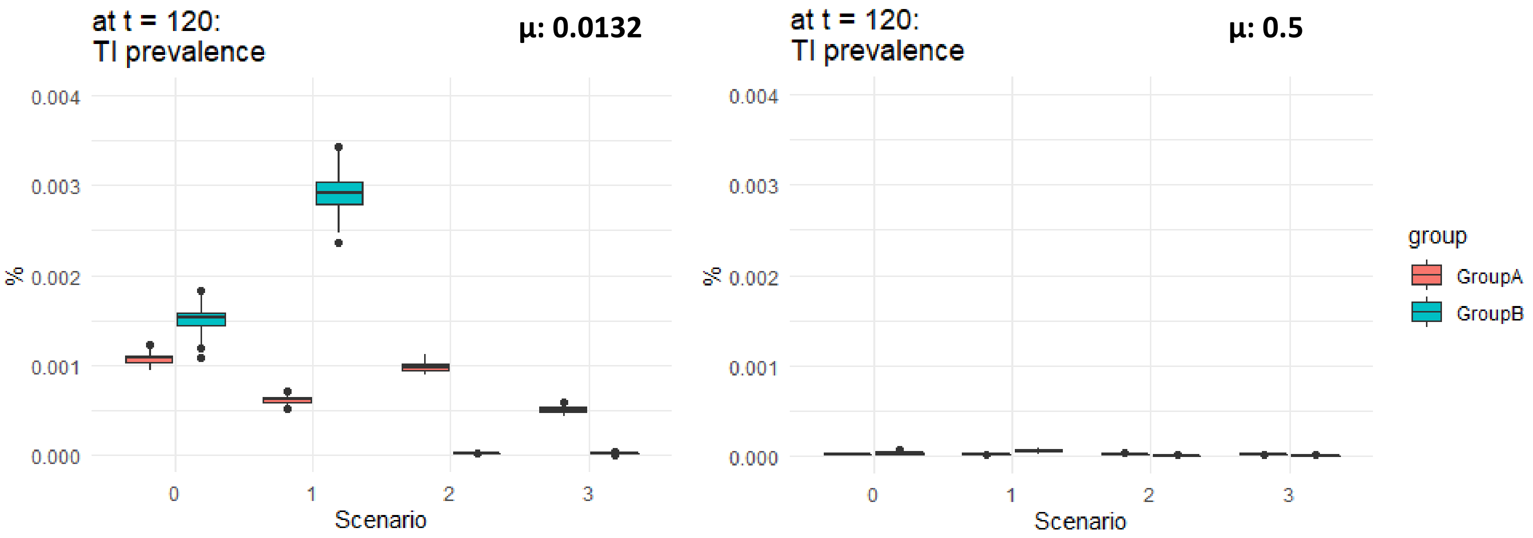

Supplementary 4. Prevalence of transiently infected animals with different µ value. The y axis presents the percentage of animals that are PI from the total cattle population in each scenario. Scenario 0 represent baseline scenario. This plot aims to compare the prevalence of PI in each type of scenario in each group at the end of simulation time ($t = 120$). Please note the y axis is slightly different from supplementary 3.

Supplementary figures: nodes degree distribution

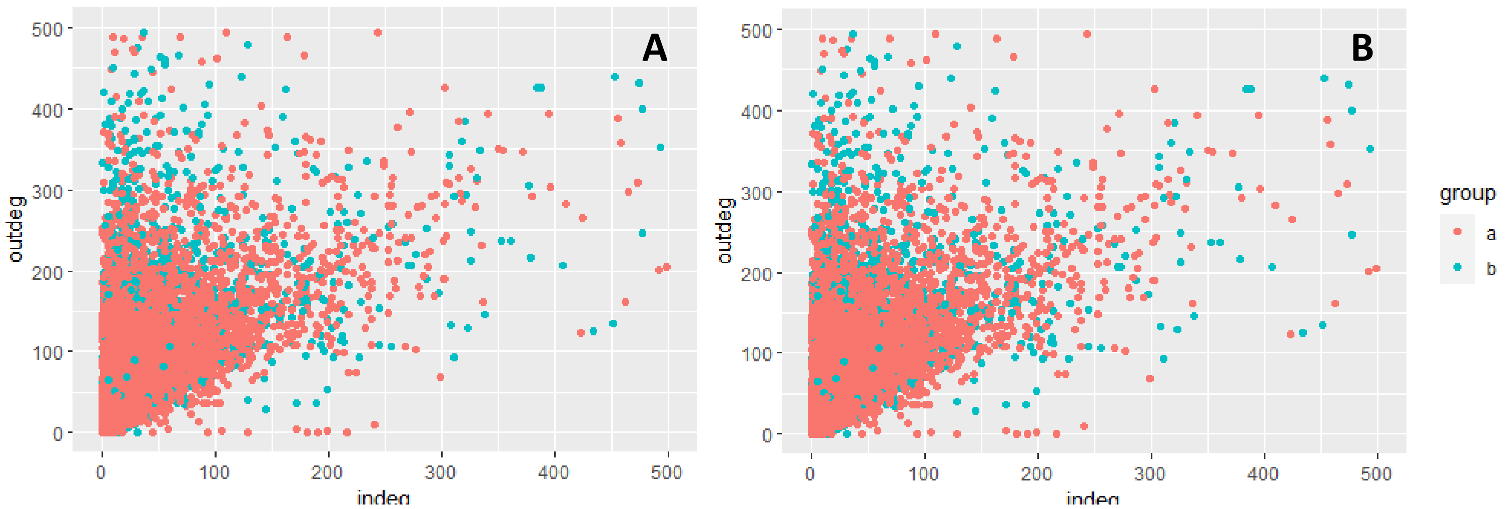

Supplementary 5. Degree distribution. This graph is showing the degree distribution of nodes that involved in animal trades in Scotland from 2008-2017. The x-axis is the node's indegree, total number of movements entering a node. While y-axis represent outdegree, the total number of movements leaving a node. Color in the graph identify in which group a node belongs to. We removed nodes that have zero indegree and outdegree. **A.** This graph is when we include all nodes movements. **B**. While in this graph, the links/movements from non-Scottish farms to farms that have 0 outdegree were removed.
